# Imaging of cartilage, meniscus, and beyond: Role of Magnetic Resonance Imaging (MRI) and Computed Tomography (CT)

**DOI:** 10.1016/j.ostima.2025.100268

**Published:** 2025-04-22

**Authors:** Patrick Omoumi

**Affiliations:** Lausanne University Hospital, Switzerland

**Keywords:** Osteoarthritis, Cartilage, Meniscus, Imaging, MRI, CT, Bone, Knee, Weight-bearing, Mri sequences, CT arthrography, Compositional imaging, Photon-counting CT

## Abstract

Magnetic Resonance Imaging (MRI) remains the reference standard for imaging cartilage and meniscus, offering superior soft tissue contrast essential for comprehensive joint assessment in osteoarthritis (OA). However, recent technological advancements in Computed Tomography (CT)—spectral imaging, and weight-bearing scanners—have sparked renewed interest in utilizing CT, and CT arthrography in the evaluation of OA. This narrative mini-review explores the strengths and limitations of both MRI and CT in imaging cartilage and meniscus, and presents some trends in the research setting.

MRI remains the modality of choice for joint imaging, offering excellent soft tissue contrast and comprehensive articular assessment. CT is the reference for the assessment of mineralized tissue imaging, and in association with arthrography (CT arthrography, CTA), provides high performance in the diagnosis of surface lesions.

In the research setting, efforts have focused on the acceleration of MRI acquisitions, with deep learning reconstructions disrupting the traditional trade-off between acquisition speed and image quality. Efforts are undertaken to standardize compositional MRI techniques, which probe early-stage biochemical tissular changes. Emerging techniques such as synthetic imaging may offer the ability to provide information on bone and soft tissues in a single acquisition. Weight-bearing acquisitions have allowed the assessment of joint structures, in particular menisci, in a loaded position. Photon-counting CT promises higher resolution, improved material separation without increasing radiation exposure. Finally, post-processing tools are being developed to leverage large quantities of data and integrate both modalities in a complementary framework that could provide a robust toolset for the assessment of OA.

## 1 Introduction

Although Magnetic Resonance Imaging (MRI) continues to be the reference standard for imaging cartilage and the meniscus, recent advancements in computed tomography (CT) imaging—such as CT arthrography, spectral imaging, and weight-bearing scanners—have reignited interest in using this technique for joint assessment. This narrative mini-review aims to explore the strengths and weaknesses of MRI and CT for the imaging of cartilage and meniscus, considering both clinical and research perspectives in the context of osteoarthritis (OA). Some emerging trends in MRI and CT research related to cartilage and meniscus imaging will also be highlighted, including MRI acceleration techniques, standardization of MRI compositional techniques, synthetic imaging, the use of weight-bearing imaging, spectral imaging, and the use of advanced automated multimodal post-processing to integrate data from different tissues.

## 2 Role of CT and MRI in clinical practice

### 2.1 MRI

In clinical practice, MRI is the established reference method for the imaging of cartilage and meniscus due to its excellent soft tissue contrast and ability to visualize the entire joint organ, which is fundamental in the context of a whole-organ disease such as OA. The performance of MRI in assessing cartilage and meniscal lesions has been extensively studied at different magnetic field strengths [[1], [2], [3], [4]]. Sensitivity to detect cartilage lesions is usually higher than specificity - in a recent meta-analysis, pooled sensitivity and specificity of 2D sequences were 76 and 93 %, respectively -, and the performance is limited for lower-grade lesions (i.e. Outerbridge grade <2) [1,2,5]. For the meniscus, MRI performance is generally good, with higher accuracy in detecting medial meniscus tears compared to lateral meniscus tears [3,4]. In a recent meta-analysis, the global sensitivity and specificity of MRI of meniscal tears were 92 % and 90 % in medial meniscal tears, and 80 % and 95 % in lateral meniscal tears, respectively [6]. Typically, tears of the posterior horn and the root of the lateral meniscus, along with ramp lesions, are commonly misdiagnosed. Some of these errors are related to technical limitations of MRI, with some tears intrinsically difficult to see at MRI, but the diagnosis of such tears is also typically considered difficult for non-expert radiologists [7].

**Fig. 1:**
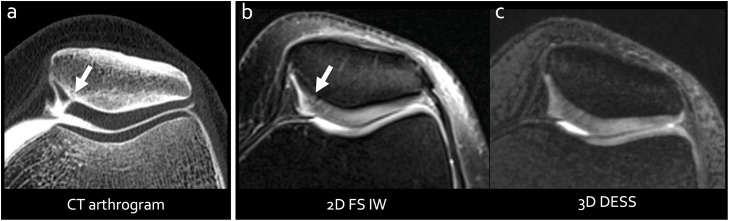
CT arthrogram (spatial resolution: 0.33, reconstructed with a slice thickness of 2 mm) (a), and corresponding 3T MR images (2D fat-saturated intermediate-weighted (2D FS IW)(acquisition parameters: spatial resolution= 0.5 × 0.5 × 3 mm, TR=3000 ms, TE=39 ms (b) and 3D Double Echo Steady State (3D DESS)(acquisition parameters: spatial resolution=0.53, TR=14 ms, TE=5 ms (c) sequences), showing down-to-bone (grade 4) cartilage defect, clearly depicted on CTA (arrow), but which only appears as signal changes on the 2D FS IW sequence and is barely visible on the 3D DESS sequence).

Moreover, it should be mentioned that not all MRI examinations are equal, and technical parameters such as acquisition time, spatial resolution, and contrast parameters must be carefully considered to optimize image quality and, consequently, diagnostic performance. For instance, while 3D DESS sequences often provide superior spatial resolution with an isotropic resolution of 0.5^3^mm, as opposed to the typical 3 mm slice thickness in 2D sequences, certain lesions are more effectively visualized in 2D when the appropriate contrast parameters are applied (Fig. 1). Although MRI protocols vary between institutions, affecting to a certain degree the performance for detecting cartilage and meniscus lesions, fat-suppressed 2D or 3D fast spin echo intermediate-weighted sequences are widely regarded as presenting the best compromise in terms of contrast between tissues of interest (Fig. 1) [5].

### 2.2 CT

**Fig. 2:**
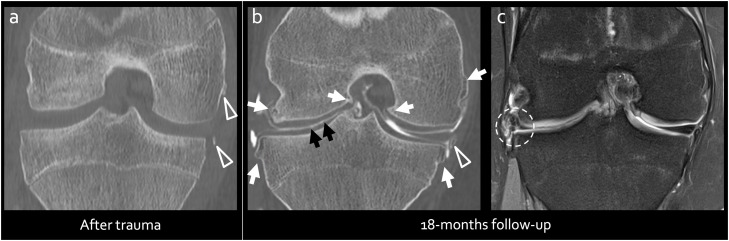
18-year-old-male after traumatic injury. Post-traumatic CT (a) shows avulsion injury at the level of meniscotibial and meniscofemoral insertions (arrowheads). Follow CT (b) showing marginal osseous proliferations around the knee (arrows), as well as the avulsed fragment which has turned into a well-delineated bony fragment (arrowhead). Subtle superficial lesions at the surface of the lateral tibial cartilage are also seen (between black arrows). These lesions are much more challenging to depict on follow-up MRI (c). Note intrameniscal degenerative signal changes in lateral meniscus (dashed circle) that do not represent a tear at CT arthrography.

In the clinical setting, CT is primarily used for its ability to assess mineralized tissues, including cortical and trabecular bone, as well as crystal deposits, thanks to its excellent contrast and spatial resolution, which can be up to 10 times higher in through-plane resolution than conventional 2D MRI sequences that typically have 3 mm slice thicknesses (Fig. 2). In the context of OA, examples of the routine uses of CT include the preoperative planning of custom-made prosthetic surgery, as well as for revision surgeries.

However, conventional CT lacks contrast for soft tissues, preventing the depiction of intraarticular structures. To address this limitation, CT arthrography (CTA) has been proposed. This technique involves acquiring a CT scan following the intra-articular injection of contrast material into the joint cavity. This technique provides great delineation of the surfaces of cartilage and menisci. Surface lesions affecting these structures (e.g., lesions communicating with the synovial cavity where the contrast material is injected) are also detected with high performance (Fig. 1, Fig. 2) [8]. CTA therefore provides an accurate assessment of cartilage and meniscal lesions in the knee, while preserving the advantages of CT for the study of mineralized tissues, such as osteophytes, subchondral bone, and crystal deposits and ossified bodies, which can be visualized with high resolution and contrast (Fig. 2).

Studies involving the knee and other joints with thinner cartilage have shown that CTA performs comparably or even superiorly to MRI in detecting surface lesions, especially when assessing lower-grade lesions (i.e. Outerbridge grade <2) (Fig. 1, Fig. 2) [9]. Additionally, CTA usually provides high diagnostic confidence due to its high contrast, high spatial resolution, and little sensitivity to motion artifacts thanks to short acquisition times. For detecting meniscal lesions, CTA has also been shown to be accurate and reproducible [10]. The sensitivity and specificity for the detection of meniscal abnormalities were found at 98 % and 94 %, respectively, with excellent interobserver agreement (kappa=0.90) [10].

In the post-operative setting, CTA has proven to be particularly useful [11]. In this context, MRI faces challenges such as metal artifacts and postoperative intrameniscal signal changes. In contrast, CTA has a lower sensitivity to metal artifacts and provides higher contrast between the intra-articular contrast material and meniscal tissue, including when compared to MR arthrography.

CTA does have some limitations, however. These include the inability to detect lesions that do not communicate with the surface, an incomplete assessment of joint structures, including the ligaments that can only partially be examined, and invasiveness [8].

## 3 Trends in the research setting

### 3.1 Faster MRI acquisitions

One of the limitations of MRI is the lengthy acquisition times, prompting significant research into the development of acceleration techniques. These efforts have led to advancements in both acquisition phase (such as parallel imaging and simultaneous multislice imaging) and in the reconstruction phase (such as compressed sensing and, more recently, deep learning reconstructions). Faster MRI acquisition times without compromising image quality are now possible. In fact, deep learning algorithms have even enabled an increase in image quality while shortening the acquisition time, eliminating the traditional trade-off between these parameters. Acceleration factors from 4 to 8 have been reported [5]. A five-minute MRI knee acquisition protocol has now become a reality in many centers, achieving similar performance than standard protocols by using a combination of methods at acquisition (parallel-imaging, simultaneous multislice imaging), or at reconstruction (compressed sensing, deep-learning reconstructions) [12].

### 3.2 Standardization of compositional MRI techniques

One key advantage of MRI compared to CT is the ability to assess biochemical changes in the chondral tissue in the early stages of disease. This is achieved through compositional MRI techniques such as T2 and T1rho mapping. T2 and T1rho values are sensitive to changes in collagen and water content, and proteoglycan concentration, respectively, and have been used in the research setting to gain insight into the early stages of cartilage degeneration. Research has shown the reliability, discriminative validity, longitudinal reproducibility as well as multicenter multivendor reproducibility of these techniques [13].

Significant efforts are being made to standardize compositional MRI techniques, with notable contributions from the Quantitative Imaging Biomarkers Alliance (QIBA) initiative of the Radiological Society of North America (RSNA) [13].

### 3.3 Synthetic imaging

One area of research focuses on the development of synthetic contrasts in MRI, which involves generating new contrasts from existing MRI data, as a method to accelerate the overall examination acquisition [14]. One application is the creation of synthetic CT images based on MRI data, with the promise to provide the complementary information typically obtained from both CT and MRI [15].

### 3.4 Use of weight-bearing

In order to assess joint structures in biomechanically more relevant conditions, weight-bearing imaging has been introduced both in MRI and CT [16,17]. These methods have provided insights into the biomechanics of menisci in particular with MRI [16], while weight-bearing CTA provides high-contrast and high-spatial resolution imaging of joint structures, most interestingly menisci, under weight-bearing conditions [17].

### 3.5 Spectral imaging

Recent years have seen regained interest for CT as an imaging technique, thanks to the advent of spectral imaging, first through dual-energy CT and more recently with photon-counting CT [18]. Compared to conventional CT, photon-counting CT offers advantages such as increased spatial resolution, and noise reduction without increased radiation exposure, as well as improved material separation. In addition to specialized applications like evaluating cartilage composition post-contrast injection and examining the role of cartilage and meniscal crystal deposits in OA, photon-counting CT may enhance both qualitative and quantitative assessments of bone [18]. Potential applications include the enhanced evaluation of subchondral mineral density and structure, offering deeper insights into the cartilage-subchondral bone unit and its involvement in OA [19].

### 3.6 Automated post-processing and multimodal imaging

Artificial intelligence has recently led to the emergence of automated post-processing tools that have opened the door to leveraging quantitative data and the spatial variations of tissue properties throughout the joint [20]. Automatic segmentation of knee structures, particularly cartilage, which has long been considered a challenging task, is now feasible due to deep learning algorithms. This advancement allows for the analysis of large volumes of data, overcoming the limitations posed by the labor-intensive nature of manual segmentation.

To maximize the wealth of information provided by 3D MRI and CT imaging data, standardized maps have been proposed as a valuable tool for studying spatial variations in tissue properties across the entire joint [20]. These maps enable the integration of data from different imaging modalities, each focusing on specific parameters such as cartilage thickness obtained through MRI, or subchondral bone mineral density obtained through CT. By utilizing these maps, researchers can develop models to explore the interrelationships between various parameters.

## 4 Conclusion

In the clinical setting, the imaging of cartilage and menisci is predominantly synonymous with MRI, a modality that also provides a whole-joint assessment. CT is primarily used as a problem-solving tool in the context of OA, particularly valued for its ability to visualize bony structures. When combined with arthrography, CT can effectively delineate surface lesions of cartilage and menisci. This feature, along with its limited sensitivity to metal artifacts, makes it especially useful for postoperative imaging.

From a research perspective, the continuous refinement of MRI and CT technologies promises further improvements in the assessment of OA in general. MRI acquisitions are made faster with high quality imaging, potentially increasing the accessibility of this modality. Compositional MRI techniques are able to detect tissular changes in the early phases of the disease and ongoing efforts aim to further standardize these methods. Synthetic CT images can be generated from MRI data, offering the potential to simultaneously provide complementary information on bone and intraarticular structures from a single examination. For CT, advancements like photon-counting technology could further enhance this modality, with enhanced spatial and spectral resolution, without increasing radiation exposure. Furthermore, post-processing tools are being developed to leverage large quantities of data and integrate both modalities in a complementary framework that could provide a robust toolset for the assessment of OA in general (Table 1).

**Table 1:** Current strengths and weaknesses of MRI and CT in the context of osteoarthritis.

Modality	Strengths	Weaknesses
**MRI**	High soft tissue contrast Non-invasive No radiation exposure Ability to obtain compositional MRI data (research setting)	Limited spatial resolution compared to CT Long acquisition time Limited visualization of mineralized tissues Less accessibility and cost than CT
**CT**	High spatial resolution Short acquisition time Excellent visualization of mineralized tissues Inherently poor soft contrast tissues, but combine with CT arthrography: excellent depiction of cartilage and menisci surface lesions Lower accessibility and cost than MRI, excellent visualization of surface lesions, shorter acquisition time	Limited soft tissue contrast (if not combined with arthrography) Radiation exposure CT arthrography: invasive (requires the intraarticular injection of contrast material)

## Declaration of competing interest

The authors declare that they have no known competing financial interests or personal relationships that could have appeared to influence the work reported in this paper.
